# Understanding variations and influencing factors on length of stay for T2DM patients based on a multilevel model

**DOI:** 10.1371/journal.pone.0248157

**Published:** 2021-03-12

**Authors:** Wen Liu, Jingcheng Shi, Simin He, Xi Luo, Weijun Zhong, Fang Yang

**Affiliations:** 1 Xiangya School of Public Health, Central South University, Changsha, China; 2 Center for Information Statistics, Health Commission of Hunan Province, Changsha, China; University of Maribor, SLOVENIA

## Abstract

**Aim:**

Shortening the length of stay (LOS) is a potential and sustainable way to relieve the pressure that type 2 diabetes mellitus (T2DM) patients placed on the public health system.

**Method:**

Multi-stage random sampling was used to obtain qualified hospitals and electronic medical records for patients discharged with T2DM in 2018. A box-cox transformation was adopted to normalize LOS. Multilevel model was used to verify hospital cluster effect on LOS variations and screen potential factors for LOS variations from both individual and hospital levels.

**Result:**

50 hospitals and a total of 12,888 T2DM patients were included. Significant differences in LOS variations between hospitals, and a hospital cluster effect on LOS variations (*t* = 92.188, *P*<0.001) was detected. The results showed that female patients, patients with new rural cooperative’ medical insurance, hospitals with more beds, and hospitals with faster bed turnovers had shorter LOS. Conversely, elderly patients, patients with urban workers’ medical insurance, patients requiring surgery, patients with the International Classification of Diseases coded complication types E11.1, E11.2, E11.4, E11.5, and other complications cardiovascular diseases, grade III hospitals, hospitals with a lower doctor-to-nurse ratio, and hospitals with more daily visits per doctor had longer LOS.

**Conclusions:**

The evidence proved that hospital cluster effect on LOS variation did exist. Complications and patients features at individual level, as well as organization and resource characteristics at hospital level, had impacted LOS variations to varying degrees. To shorten LOS and better meet the medical demand for T2DM patients, limited health resources must be allocated and utilized rationally at hospital level, and the patients with the characteristics of longer LOS risk must be identified in time. More influencing factors on LOS variations at different levels are still worth of comprehensive exploration in the future.

## Background

Diabetes mellitus is one of the fastest-growing chronic and non-communicable diseases in the 21^st^ century. The consumption of medical resources and demand for health services by diabetes mellitus patients have placed great pressure on most countries’ public health systems [1, 2]. Among these diabetes mellitus are type 2 diabetes mellitus (T2DM), accounting for over 90% of all diabetes mellitus types [2]. How to deal with the challenges stemming from the increasing number of T2DM patients and make full use of existing medical resources to better meet their medical demand is a common and urgent issue faced by most countries in the public health field.

Previous attempts at exploratory research have suggested that shortening the length of stay (LOS) could be a potential and sustainable way to ease this foreseeable problem [3–6]. LOS is a sensitive indicator that reflects the disease burden of patients, as well as resource utilization and service quality for hospitals. The average LOS of inpatient in China is still much higher than countries with much more highly-developed medical standards, such as the United States and Australia [7]. Prolonging LOS not only occupies beds and caregivers for a longer time, leading to poor utilization of medical resources and increasing the workload of medical staff, but more seriously, it also stimulates the occurrence of adverse events in hospitals, such as infections [8–10] and other complications unrelated to admission diagnosis [11, 12]. Conversely, shortening LOS sharply reduces patients’ costs and more hospitalization needs can be met. Now, LOS is regarded as a key indicator for the evaluation of hospitals’ service quality and is an essential parameter for health reports on many occasions in terms of inpatient management and evaluation [13]. However, the influencing factors that affect LOS variations for T2DM patients remain unclear. Ways of effectively reducing LOS for T2DM patients have also not been comprehensively discussed.

Several measures and efforts have been taken to reduce LOS [12, 14–16]. Most of those efforts simply focused on the characteristics of the patient and disease at individual level [17–20]. Furthermore, some research projects on certain diseases have suggested that a non-negligible part of LOS variations can be explained by the characteristics of organization and resource at hospital level [14, 21, 22]. As the medical care provided by one hospital is similar for all patients with the same clinical situation, the outcome for these patients varies slightly at the same hospital but quite significantly at different hospitals. The adjustment effect of hospital’ organization and resource on outcome variables is known as hospital cluster effect [23].

Therefore, it is assumed that the influencing factors that affect LOS variations for T2DM patients can be attributed to either patient and disease features at individual level [24] or organization and resource features at hospital level. A multilevel, multivariable linear regression analysis was adopted to test the hospital cluster effect for LOS variations, screen the potential influencing factors of LOS variations, and evaluate the relative importance of these factors. All the work we have done is expected to provide some guidance for reducing LOS in T2DM patients.

## Materials and methods

### Data source

For this research, all data was obtained from the Straight-Line Reporting System (SLRS) dataset affiliated with the Health Commission of Hunan Province, China. Information was collected as the basis for managing and evaluating the medical service from two aspects: patients’ electronic medical records, including diagnosis and treatment details, admissions and discharges, etc; and hospitals’ regular record, including organization type and code, professional classification (general or specialized), hospital grade (grade I, II, or III), the number of beds and staff, etc. All data must comply with regulations on the administration of health statistics in the SLRS dataset issued by the Ministry of Health of the People’s Republic of China. According to these regulations, the connection between each patient’s electronic medical records and the hospitals’ organization and resource features can be linked by the name and organization code of each hospital.

The SLRS database is completely protected and is unavailable to any researchers or individuals without authorization. As the number of inpatients in the SLRS database is accumulated at a rate of tens of millions per year, some statistical information calculated from the electronic medical records in the SLRS database has proved an important reference value for health policy decisions. The Health Commission of Hunan Province has gradually made part of the data available to some reliable institutions for exploring the potential value under the condition of anonymous and intranet. The Central South University, where we are, is one of few scientific research institutions to be accredited by the Health Commission of Hunan Province, and our project has also been approved by the Ethical Committee of Xiangya School of Public Health Central South University with the approval number XYGM-2020-99.

### Sample selection

A multi-stage random sampling was chosen for capturing the targeted T2DM patients from specific hospitals. For multilevel model analysis, the number of groups is more important than a larger number of units within each group [25]. Maas & Hox [26] discovered that a minimum of 50 groups is required for obtaining unbiased parameter estimates of standard errors and statistical power. Therefore, 50 were chosen as the hospital level sample size and selected as the primary sampling groups from general hospitals where more than 500 patients with T2DM were discharged in 2018. Considering the complexity of the SLRS dataset and the large gap in the number of T2DM patients in each hospital, only 20% of T2DM patients’ electronic medical records were then randomly chosen from each primary sampling group as the final sample [23].

LOS refers to the number of days that patients spend in hospital, and this figure was recorded on the first page of the electronic medical record. Patients whose LOS was more than 1 day between January 1, 2018 and December 31, 2018, were older than 18 years old, were principally diagnosed with T2DM, had complete admission records, and were discharged with doctor approval were included. Compared with the recalculated LOS on the admission and discharge date, patients with a difference of more than 1 day in LOS do not represent the sample of this study. We also excluded patients transferred from other hospitals, whose hospitalization costs equal to or close to zero, and the discharge method was recorded as “death”.

Potential influencing factors that affect LOS variations in the individual and hospital levels were chosen and divided into the following three categories: 1) Patient-related variables: age, gender, marital status, medical insurance, admission method, whether or not they have had an operation; 2) Complication-related variables: the classification of complication types refer to the International Classification of Diseases (version-10) (ICD-10) codes, which includes E11.1 (with acidosis complications), E11.2 (with kidney complications), E11.3 (with ophthalmic complications), E11.4 (with neurological complications), E11.5 (with circulatory complications), and other complication types includes abnormal blood pressure (ABP) and cardiovascular diseases (CVDs) defined as a diagnosis of coronary heart disease, heart failure, arrhythmia, myocardial infarction [27]; 3) Hospital-related variables: grade, beds, doctor-to-nurse ratio, daily visits per doctor, and bed turnovers. Factors at hospital level include hospital-related variables, while at individual level include patient-related and complication-related variables.

A series of data cleaning processes were utilized after sampling. Missing age and gender information were completed based on citizen identity cards, where the 7-14th digits denote the date of birth. An odd number in the 17th digit indicates a male, and an even number in the 17th digit indicates a female. The value of LOS was examined using admission and discharge data, and the consistency of the disease name with the ICD-10 codes was also rechecked.

## Methods

A multilevel model was used for testing the hypothesis of LOS variations with hierarchy structure, equivalent to hospital cluster effect on LOS variations, and to screen the influencing factors of LOS variations for T2DM patients. Usually, a multilevel model is generally applied to data with the hierarchical structure where lower level (individual level) units are nested in higher level (hospital level) groups, and the feature of higher level groups influences the outcome variable of lower level units. Furthermore, the total variance for outcome variable can be classified as between-group variance and within-group variance. For multilevel analysis, fitting an “empty model” is the initial step, and this does not include any explanatory variables but simply considers whether the hierarchy structure or hospital cluster effect on LOS variations exists. The intraclass correlation coefficient (*ICC*) [28] is defined as the ratio of between-group variance to the total variance ranging from 0 to 1. A lower *ICC* indicating a smaller hospital cluster effect on LOS variations for T2DM patients. The proportional reduction of mean squared prediction error (*PR*) [29] proposed by Snijders and Bosker could quantify the interpretability degree of the explanatory variable on the outcome at various levels based on the between-group and within-group effects.

Besides, the multilevel linear regression model attached greater importance to the outcome variable follows a normal distribution [28]. To better apply to the model, we took a box-cox transformation to normalize LOS [30]. The box-cox transformation parameter *λ* approached 0 (*λ* = 0.043) in our data, which was equivalent to a natural logarithmic transformation. Logarithmic transformation on the outcome variable to mitigate the effects of non-normal distribution is widely applied in research practice. For data with extremely skewed distribution, such as LOS, even if the transformation finally still does not meet the standard normal distribution, the approximate normal distribution is also acceptable for multilevel model analysis [31–33].

### Statistical analysis

The distribution of the LOS was negative skewness. Therefore, the median (*M*) and quartile range (*QR*) were used for summarizing the distribution feature of LOS by hospital. A histogram displays the frequency distribution of LOS for all T2DM patients. Descriptive variables were summarized using the mean and standard deviation for quantitative variables and the relative frequencies for categorical variables. Continuous variables were processed using grand mean centering to increase the model’s rapid convergence and reduce the multicollinearity.

Multilevel empty models and *ICC* were utilized for testing whether the hospital cluster effect on LOS variations exist. The relationships between LOS and patient-related variables and complication-related variables at individual level were tested by Mann-Whitney U test for binary variables and Kruskal-Wallis H test for unordered categorical variables in univariate analysis. Both variables at individual level with statistical significance in univariate analysis and hospital-related variables at hospital level were included in the multilevel, multivariate linear regression analysis to build the “final model” with transformed LOS as an outcome variable. *PR* was used for measuring the interpretable degree of explanatory variables to LOS variations at various levels. Likelihood ratio test (LR) and Akaike information criterion (*AIC*) were used for the evaluation of the fitting ability of these models. *P* values < 0.05 were considered as statistical significance. All statistical analyses were performed using R version 3.6.3. The main packages used in the analysis were “lme4”, “matrix”, and “lattice”.

## Results

### Summary of LOS variations in T2DM patients

Based on multi-stage random sampling, 50 hospitals and a total of 12,888 individuals discharged with T2DM were included in the analysis. The summary of all the patients’ LOS variations by hospital was shown in Table 1. The number of T2DM patients at each hospital ranged from 106 to 626. There were significant differences in LOS variation between hospitals, and the maximum median was 12d (H28), while the minimum median was 6 d (H36, H43, H44). The median of LOS for all T2DM patients was 9 d (*QR* 7–11 d).

**Table 1:** Summary of patients’ LOS variations by hospital.

HospID	*N*	*M*	*QR*	HospID	*N*	*M*	*QR*
**H1**	626	9	8–12	**H26**	240	8	7–11
**H2**	505	9	7–11	**H27**	227	11	8–15
**H3**	495	10	8–12	**H28**	206	12	8–15
**H4**	493	9	7–12	**H29**	202	9	6–13
**H5**	459	8	6–11	**H30**	179	7	6–10
**H6**	406	9	8–11	**H31**	179	10	9–12.5
**H7**	387	9	7–11	**H32**	170	7	5–9
**H8**	369	8	6–11	**H33**	170	8	5.25–12
**H9**	366	7	5–9	**H34**	168	11	8–17
**H10**	355	9	7–12	**H35**	167	11	8–14
**H11**	345	8	7–11	**H36**	165	6	5–8
**H12**	341	7	6–9	**H37**	162	7	5–9
**H13**	320	8	7–10	**H38**	158	8	6–10
**H14**	316	8	7–10	**H39**	157	8	5–11
**H15**	307	8	6–10	**H40**	144	10.5	8–15
**H16**	307	9	8–12	**H41**	136	7	6–10
**H17**	305	11	8–14	**H42**	135	10	7–12.5
**H18**	301	10	8–13	**H43**	134	6	4–7
**H19**	297	10	7–12	**H44**	132	6	5–8
**H20**	295	8	6–10	**H45**	131	7	5–10
**H21**	283	8	6–11	**H46**	129	9	7–13
**H22**	277	8	7–10	**H47**	128	8	6–11
**H23**	267	11	9–14	**H48**	128	8	6–11
**H24**	254	8	6–10	**H49**	120	8	6–9
**H25**	243	9	7–12	**H50**	102	8	6–10
**All**	12888	9	7–11				

**Fig 1:**
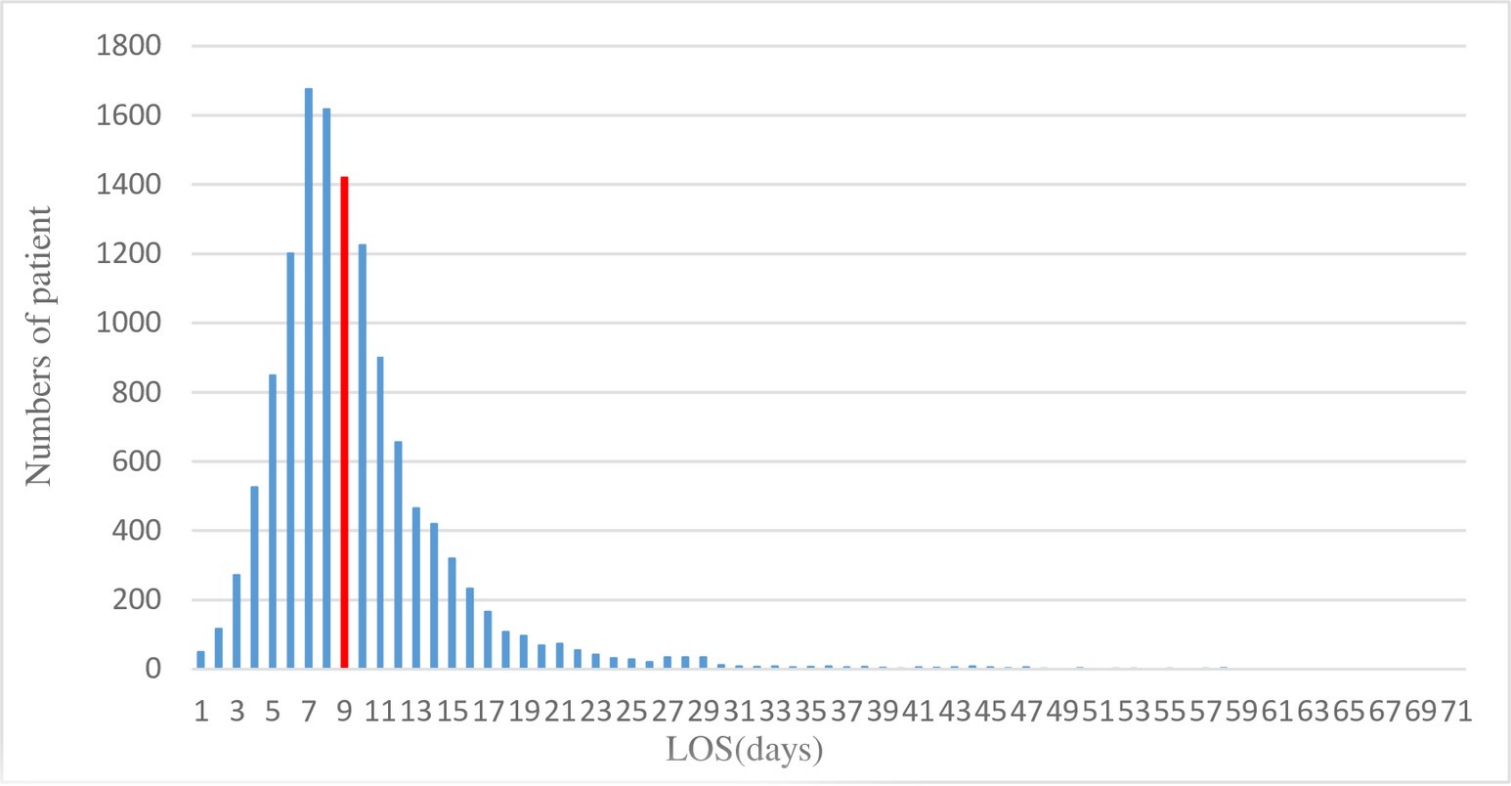
Numbers of patients by LOS.

As shown in Fig 1, the frequency distribution diagram indicated that LOS had a negative skewness distribution with a skew coefficient (6.75) and kurtosis coefficient (114.59). Following a natural logarithmic transformation to LOS, both skew coefficients (-0.08) and kurtosis coefficients (2.58) decreased dramatically, approaching a normal distribution.

### Testing the assumption of hierarchy

In order to test the assumption of LOS variations with hierarchy structure, the multilevel empty models comparing variance at hospital level and total variance showed statistical significance (*AIC* = 17333, *t* = 92.188, *P*<0.001). What’s more, *ICC* was calculated to be 10.5% (0.026/(0.026+0.222)), indicating the clustering effect can explain the 10.5% LOS variations at hospital level, as presented in Table 2. By combining the above points, there is overwhelming evidence that the hospital cluster effect on LOS variations cannot be ignored.

**Table 2:** Summary of multilevel empty model.

Parameter	*b*	$S_{\bar{b}}$	*t/Wald Z*	*P*	*OR(95% CI)*	
					lower	upper
**Fixed effects**						
Intercept	2.133	0.023	93.14	<0.001	2.087	2.180
**Random effects**						
Hospital level	0.026	0.005	4.718	<0.001	0.017	0.039
Individual level	0.222	0.003	80.116	<0.001	0.216	0.227

An LR test was conducted which compared the empty model of multilevel (hospital level and individual level) with single level (individual level). Statistical significance was detected between these models (*-2LL* = 917.94, *P*<0.001). Therefore, multilevel model is more suitable than the single level model when analyzing the variation and influencing factors of LOS for T2DM patients. It is reasonable to consider the variables at hospital level when analyzing the influencing factors on LOS variations through a multilevel model.

### General description and univariate analyses of LOS variations for T2DM patients

A general description of the patients and hospitals was summarized in Table 3. The majority of T2DM patients were male (53%, n = 6,826) and over 60 years old (52%, n = 6,752). 12,635 patients (98%) were married, 4,410 patients (34%) were urban workers insurance. 10,554 patients (82%) admission method were outpatient. Only 552 patients (4%) had surgery. The most common complications of T2DM patients were CVDs, ABP, and E11.4, with the patients number 4,755 (37%), 4,542 (35%), and 4,540 (35%), respectively. 26 of the 50 hospitals were rated as grade III, and the average doctor-to-nurse ratio was 1:1.66. The mean and standard deviation of the number of beds, daily visits per doctor, and bed turnovers were 1373.3 and 747.4, 5.6 and 2.3, 41.1 and 8.5, respectively.

**Table 3:** General description and univariate analyses of LOS variations for patients.

Variables	Total sample *N(%)/M±SD*	Length of stay	
		*M*	*QR*
**Patient-related variables**			
Age*			
18–40	614(0.05)	8	6–11
41–60	5522(0.43)	8	6–11
≥61	6752(0.52)	9	7–12
Gender*			
Male	6826(0.53)	9	7–12
Female	6062(0.47)	8	7–11
Marriage			
Married	12635(0.98)	9	7–11
Unmarried	253(0.02)	9	7–12
Medical insurance*			
Urban workers	4410(0.34)	9	7–12
Urban residents	2991(0.23)	8	6–10
New rural cooperative	3612(0.28)	8	6–10
Other	1875(0.15)	9	7–12
Admission method*			
Outpatient	10554(0.82)	9	7–11
Emergency	2334(0.18)	8	6–11
Operation*			
No	12336(0.96)	9	7–11
Yes	552(0.04)	11	7–18
**Complication-related variables**			
E11.1*			
No	12383(0.96)	9	7–11
Yes	505(0.04)	9	7–11
E11.2*			
No	9924(0.77)	8	6–11
Yes	2964(0.23)	9	7–12
E11.3*			
No	10262(0.80)	8	7–11
Yes	2625(0.20)	9	7–12
E11.4*			
No	8348(0.65)	8	6–11
Yes	4540(0.35)	9	7–11
E11.5*			
No	11417(0.89)	8	7–11
Yes	1471(0.11)	10	8–13
ABP*			
No	8346(0.65)	8	7–11
Yes	4542(0.35)	9	7–12
CVDs*			
No	8133(0.63)	8	6–11
Yes	4755(0.37)	9	7–12
**Hospital-related variables**			
Hospital-grade			
Grade II	24(0.48)	8	6–10
Grade III	26(0.52)	9	7–12
Doctor-to-nurse ratio	1:1.66	—	—
Number of beds	1373.3±747.4	—	—
Daily visits per doctor	5.6±2.3	—	—
Number of bed turnovers	41.1±8.5	—	—

*M±SD*: mean *±* standard deviation; E11.1: with acidosis complications; E11.2: with kidney complications; E11.3: with ophthalmic complications; E11.4: with neurological complications; E11.5: with circulatory complications;

‘*’: Univariate analysis *P*<0.05.

The univariate analyses for the influencing factors with LOS variations at individual level were summarized in Table 3. It is indicated that all complication-related variables and other patient-related variables, except for marital status, had statistical significance with LOS variations(*P*<0.05).

### Models and influencing factors for LOS variations

Firstly, patient-related variables and complication-related variables at individual level with statistical significance in the univariate analysis were added as covariates to multilevel empty models. *AIC* then dropped from 17,333 to 16,764, and the LR test comparing the empty model and model with variables at individual level was statistically significant (*-2LL* = 597.47, *P*<0.001).

Secondly, variables at hospital level following grand mean centering were also added as covariates to the multilevel models with variables at individual level. *AIC* then dropped from 16,764 to 16,747, and the LR test comparing the models with variables at individual level and models with variables at both levels was statistically significant (*-2LL* = 26.967, *P*<0.001). *PR* calculated by variance change of these two models showed that the hospital level’s explanatory variables in the final model included variables at both levels, accounted for 18.2% of LOS variations at hospital level and 2.3% at individual level.

Finally, the multilevel, multivariate regression results showed that female patients, patients with new rural cooperative’ medical insurance, hospitals with more beds, and hospitals with a faster bed turnover had shorter LOS, whereas elderly patients, patients with urban workers’ medical insurance, patients who required surgery, patients with ICD-10 coded complications types E11.1, E11.2, E11.4, E11.5 and other complications CVDs, grade III hospitals, hospitals with a lower doctor-to-nurse ratio, and hospitals with more daily visits per doctor had longer LOS, as presented in Table 4.

**Table 4:** Multilevel, multivariate analysis of LOS variations for patients.

Parameter	*b*	$S_{\bar{b}}$	*t*	*P*
**Fixed effects:**				
Intercept	1.751	0.114	15.316	<0.001
Gender (reference = male)	-0.02	0.008	-2.395	0.017
Age^※^	0.003	0.000	6.854	<0.001
Medical insurance (reference = other)				
Urban workers	0.054	0.015	3.731	<0.001
Urban residents	-0.02	0.016	-1.279	0.201
New rural cooperative	-0.053	0.016	-3.305	0.001
Emergency (reference = outpatient)	0.006	0.015	0.382	0.703
Operation	0.314	0.021	14.900	<0.001
E11.1	0.066	0.022	3.010	0.003
E11.2	0.064	0.010	6.200	<0.001
E11.3	-0.005	0.011	-0.476	0.634
E11.4	0.034	0.010	3.538	<0.001
E11.5	0.111	0.014	7.849	<0.001
ABP	3.89e^-04^	0.009	0.043	0.966
CVDs	0.05	0.009	5.338	<0.001
Hospital grade (reference = Grade II)	0.134	0.043	3.131	0.003
Doctor-to-nurse ratio^※^	0.003	0.001	2.131	0.038
Number of beds^※^	-8.21e^-05^	2.94e^-05^	-2.792	0.008
Daily visits per doctor^※^	0.025	0.009	2.641	0.011
Number of bed turnovers^※^	-0.007	0.002	-3.023	0.004
**Random effects:**				
Hospital level	0.0104	0.1020	—	—
Individual level	0.2119	0.4603	—	—

E11.1: with acidosis complications; E11.2: with kidney complications; E11.3: with ophthalmic complications; E11.4: with neurological complications; E11.5: with circulatory complications; ‘※’: Grand mean centering.

## Discussion

Chronic disease generally requires longer LOS than other diseases in most areas and hospitals. The average LOS of T2DM patients in this study was 9.8 d, slightly higher than 9.3 d represented the average LOS for all hospitalized diseases in China in the same period [13]. However, it was much higher than other countries with higher-quality medical services, such as Australia and Ireland [7]. Therefore, shortening LOS is a potential way to reduce the pressure and better meet the growing medical needs of T2DM patients with limited resources.

Previous studies have focused more on the relationship between LOS and characteristics of patients or disease at individual level, and have ignored the adjustment effects of organizational structure and resource distribution at hospital level [14, 20, 22]. These efforts cannot effectively reduce LOS caused by factors other than disease and patient characteristics. In this research, an in-depth exploration of the relationship between LOS variations and potential factors from three categories at two levels was conducted, including patient-related factors and complication-related factors at individual level, hospital-related factors at hospital level. As far as we know, no research has ever studied the effects of organization and resource on LOS variations for those admitted with T2DM in the Chinese healthcare system.

Female patients, patients with new rural cooperative’ medical insurance had shorter LOS, whereas elderly patients, patients with urban workers’ medical insurance, patients who required surgery had longer LOS. Male patients and elderly patients are in worse condition, and longer LOS is necessary [23, 34, 35]. Lagoe et al. concluded that the shift to reimbursement by discharges initiated by medical insurance was a major cause of LOS reduction [36]. Patients with medical insurance have longer LOS than those without [16, 22]. The different medical insurance types also lead to patients’ different medical behavior, including the choice of hospital, LOS, and cost [4]. Similar results were also found in our research. Patients with new rural cooperative’ medical insurance have shorter LOS, while urban workers medical insurance receives longer LOS. Besides, patient who need an operation require more hospitalization time as disease condition is generally more severe. Moreover, they are susceptible to adverse events after surgery [19]. This is a particular objective fact for diabetes patients [20, 37].

Patients with ICD-10 coded complications types E11.1, E11.2, E11.4, E11.5 and other complications CVDs had longer LOS. Moreover, E11.5 had the greatest effect on LOS variations. Few researchers have focused on the relationship between LOS and diabetes-related complications classified by ICD-10 codes. Although the ICD-10 codes classification problem may exist [38–40], ICD-10 codes have a good distinction between diabetes and its complications [41]. Considering the ICD-10 codes’ importance in clinical practice, our research attempts to explore the relationship between LOS variations and ICD-10 coded complications for T2DM patients. Similar results from Bader et al. showed that comorbidities and complications worsen patient’s conditions and lead to longer LOS [42]. Nawata et al. found that E11.5 and E11.7 (multiple complications) have a positive impact on LOS variations [30, 34]. Unanimous conclusions were reached by Cheng et al. CVDs are one of the most common complications of diabetes admissions and are always accompanied by a relatively longer LOS [17].

All hospital-related variables in this study were statistically significant for LOS variations. Grade III hospitals and daily visits per doctor had the most significant impact on LOS variations. Hospital-grade is a comprehensive index in China that evaluates hospital’s qualification based on its scale, scope of services, and medical equipment. Generally, grade III hospitals with larger scale and more advanced equipment can provide a wider range of diagnostic and therapeutic services for cases, especially for patients whose condition has worsened and need better care, such as referral patients. Therefore, the LOS of T2DM patients in grade III hospitals is usually longer. Beds, doctors, and nurses are the core medical resources in health institutions, and their number and distribution directly impact the normal running of medical service. A lack of these medical resources can dramatically reduce service efficiency and increase LOS [43]. Although the number of beds per thousand people in China has gradually increased in recent years [44], compared to the scale of beds in developed areas, beds are playing an important role in LOS variations and are still in demand. The doctor-to-nurse ratio was 1:1.66 in this study, compared to the global average of 1:2, and even some developed areas such as Japan and the United States reached 1:4 [43, 44]. Thus it also has space for the number of doctors and nurses to increase. Not only is there a shortage of medical staff [43], but high-quality medical professionals are urgently required, which is closely related to the reduction of LOS [15, 45]. Daily visits per doctor and bed turnovers are indicators associated with staff workload and resource utilization [46]. A higher number of daily visits per doctor means a heavier workload for doctors, and less time and energy for providing better medical service to patients, especially for T2DM patients who need long-term care to control blood sugar. Bed turnovers measures the average number of patients discharged from each bed during a certain period. Measures have been implemented to speed up the bed turnovers for emergency cases [47]. Faster bed turnover makes more beds available for other patients, in other words, each patient’s LOS is relatively shortened. However, whether these measures will also be feasible for reducing LOS in T2DM patients remains unclear.

Finally, it must be admitted that variables chosen from the SLRS database were already established, other variables outside of the SLRS database were unable to be included in this study. Further studies on the LOS variations and factors for T2DM patients should be conducted from comprehensive perspectives, such as income level, dietary and environmental factors.

## Conclusion

This study analyzed the LOS variations and influencing factors for T2DM patients using a multilevel linear regression model. A significant difference in LOS variation of T2DM patients was detected across hospitals in Hunan, China. Evidence had also proved that hospital cluster effect on LOS variations did exist. Moreover, the results showed that complication and patient characteristics at individual level, as well as organization and resource characteristics at hospital level, had impacted LOS variations to varying degrees.

To shorten LOS and better meet the medical demand for T2DM patients, we suggest that limited medical resources must be allocated and utilized rationally at hospital level, and patient characteristics with longer LOS risk must be identified in time. More influencing factors on LOS variations from different levels are still worth of comprehensive exploration in the future.

## Supporting information

S1 File (CSV)
